# Decoding Breast Cancer: Using Radiomics to Non-Invasively Unveil Molecular Subtypes Directly from Mammographic Images

**DOI:** 10.3390/jimaging10090218

**Published:** 2024-09-04

**Authors:** Manon A. G. Bakker, Maria de Lurdes Ovalho, Nuno Matela, Ana M. Mota

**Affiliations:** 1 Faculty of Science and Engineering, University of Groningen, 9700 AS Groningen, The Netherlands; 2 Departamento de Radiologia, Hospital da Luz Lisboa, Luz Saúde, 1500-650 Lisboa, Portugal; 3 Instituto de Biofísica e Engenharia Biomédica, Faculdade de Ciências, Universidade de Lisboa, 1649-004 Lisbon, Portugal; 4 Departamento de Física, Faculdade de Ciências, Universidade de Lisboa, 1649-004 Lisbon, Portugal

**Keywords:** breast cancer, molecular subtypes, radiomics, mammography, support vector machine, naive Bayes, machine learning

## Abstract

Breast cancer is the most commonly diagnosed cancer worldwide. The therapy used and its success depend highly on the histology of the tumor. This study aimed to explore the potential of predicting the molecular subtype of breast cancer using radiomic features extracted from screening digital mammography (DM) images. A retrospective study was performed using the OPTIMAM Mammography Image Database (OMI-DB). Four binary classification tasks were performed: luminal A vs. non-luminal A, luminal B vs. non-luminal B, TNBC vs. non-TNBC, and HER2 vs. non-HER2. Feature selection was carried out by Pearson correlation and LASSO. The support vector machine (SVM) and naive Bayes (NB) ML classifiers were used, and their performance was evaluated with the accuracy and the area under the receiver operating characteristic curve (AUC). A total of 186 patients were included in the study: 58 luminal A, 35 luminal B, 52 TNBC, and 41 HER2. The SVM classifier resulted in AUCs during testing of 0.855 for luminal A, 0.812 for luminal B, 0.789 for TNBC, and 0.755 for HER2, respectively. The NB classifier showed AUCs during testing of 0.714 for luminal A, 0.746 for luminal B, 0.593 for TNBC, and 0.714 for HER2. The SVM classifier outperformed NB with statistical significance for luminal A (*p* = 0.0268) and TNBC (*p* = 0.0073). Our study showed the potential of radiomics for non-invasive breast cancer subtype classification.

## 1. Introduction

Breast cancer accounts for 32% of all new cancer diagnoses in 2024, making it the most commonly diagnosed cancer in the world [1]. Among women, it accounts for 7% of cancer deaths, surpassing lung cancer as the primary cause of cancer mortality [2]. To decrease this high mortality rate, early detection and diagnosis are of high importance. Numerous countries worldwide have breast cancer screening programs in which digital mammography (DM) is the gold standard imaging [3]. To confirm the diagnosis of a suspected lesion on DM, a core needle biopsy (CNB) is performed. This provides crucial histological information regarding the tissue, necessary for classifying the breast cancer type and tumor grade [4]. Based on the immunohistochemical (IHC) expression of hormone receptors and the Ki67-antigen obtained from a CNB, breast cancer can be divided into four different subtypes. The most commonly used subtypes of breast cancer are luminal A (estrogen receptor positive (ER+), progesteron receptor positive (PR+) and human epidermal growth factor receptor negative (HER2−)), luminal B (ER+, PR+/−, and HER2−), HER2-positive (ER−, PR−, and HER2), and triple-negative breast cancer (ER−, PR−, and HER2−). The Ki-67 antigen is a marker for cancer proliferation. Classifying these subtypes and obtaining their Ki-67 antigen reveals a strong association with variations in tumor aggressiveness, therapeutic response, and prognosis [4,5,6]. Accurately classifying these subtypes is therefore crucial for guiding treatment decisions and improving patient outcomes. Despite the benefits, CNB has its drawbacks. The risks associated with the examination include bleeding, hematoma, and infection [7]. Patients may also experience pain during the procedure, and initial samples can be inconclusive, leading to additional biopsies. Obtaining the additional samples can be complicated due to previously induced changes at the biopsy site, potentially influencing the histopathologic evaluation. Furthermore, CNB is a technique that is expensive and requires highly specialized resources for both collection and analysis. These resources can be very scarce in less developed countries. However, the main limitation during CNB is the limited sample size. Since the biopsy does not represent the entire heterogeneity of the tumor volume, it does not capture the full extent of the tumor [4]. To address these challenges, non-invasive techniques, such as liquid biopsy, specifically circulating tumor DNA (ctDNA) analysis, and circulating tumor cells (CTCs), have emerged as promising alternatives. ctDNA allows for the detection of tumor-specific genetic alterations through a blood sample [8,9]. This offers a less invasive method for the screening, real-time monitoring treatment response, and evaluation of disease progression compared to CNB. However, ctDNA analysis has limitations, particularly in early stages of breast cancer, where its sensitivity is relatively low compared to many other tests. Additionally, the detection rate varies by breast cancer subtype [8]. A more accurate classification of the subtypes can significantly contribute to advanced cancer treatments and improved clinical outcomes. Radiomics plays an important role in addressing the constraints of the CNB and other liquid biopsy techniques. It involves extracting quantitative, tumor-specific information from medical images that is not visible to the human eye [10,11]. Unlike CNB or ctDNA, radiomics can derive information about heterogeneity from an entire region of interest (ROI) [12]. Additionally, the extraction of tumor region information directly from the DM images makes this technique more cost-effective and accessible, particularly in less developed countries.

Several studies have shown that radiomics extracted from contrast-enhanced spectral mammography (CESM) shows the potential for non-invasively predicting breast cancer subtypes [13,14,15,16,17]. In CESM, the injection of a contrast agent makes it an invasive procedure, posing the risk of allergic reactions in patients. Furthermore, CESM is not the gold standard for breast cancer screening. Therefore, it is of high importance to explore the potential of using DM images for predicting breast cancer subtypes. Previous studies have demonstrated the capability of predicting breast cancer subtypes based on radiomic features extracted from DM images [18,19,20,21,22]. Table 1 provides a summary of these studies, highlighting their investigations into breast cancer subtype prediction through radiomics. Many existing studies rely on CESM as the imaging technique, involve small datasets, or focus on predicting a single breast cancer subtype. In contrast, our study aims to predict all molecular subtypes of breast cancer using a large database and DM images, which is a widely available and the current gold standard imaging technique.

The purpose of this study was to investigate the potential of predicting the molecular subtypes of breast cancer with the use of machine learning (ML) and radiomic features extracted from screening DM images.

## 2. Materials and Methods

### 2.1. Database

A retrospective study based on the OPTIMAM Mammography Image Database (OMI-DB) was performed [24]. This dataset contains screening DM images and patient data from the United Kingdom breast cancer screening program within a time period of 2011 until May 2020. The entire database included information from 173,000 women, of which 10,000 were normal cases; 5,500 and 800 were marked and unmarked as malignant, respectively; and 600 and 1000 were benign marked and unmarked, respectively. Based on the hormone receptor status available in the database, the molecular subtypes were determined and classified into groups (Table 2): luminal A, luminal B, TNBC, and HER2. Furthermore, the database contained detailed image annotations made by expert radiologists regarding the tumor regions. These marks were used to provide accurate reference points for the tumor locations. Images from different mammography equipment manufacturers were included. The equipment manufacturers used was predominantly Hologic (Bedford, MA, USA) (384 images), followed by Siemens (Siemens AG, Healthcare Sector, Erlangen, Germany) (14 images), GE (Madison, WI, USA) (8 images), and Sectra (Sectra AB, Linköping, Sweden) (7 images). The images all had 16-bit quantization with varying pixel sizes ranging from 0.0625 × 0.0652 mm to 0.1 × 0.1 mm.

### 2.2. In- and Exclusion Criteria

**Figure 1:**
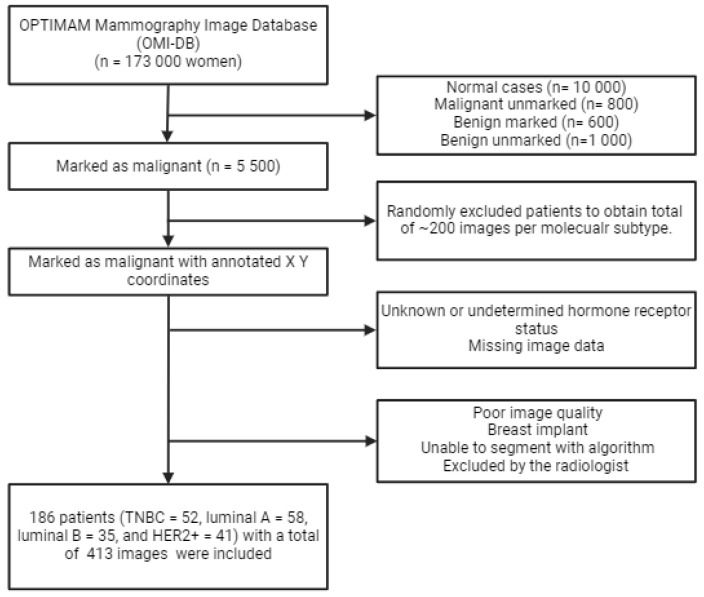
The in- and exclusion criteria flowchart used during this study.

A flowchart of the in- and exclusion criteria is depicted in Figure 1. During this study, all types of lesions, including masses, calcifications, architectural distortions, and asymmetries, were included. The inclusion criteria for the study were as follows: (I) malignantly proven DM images; (II) determined hormone receptor status; and (III) annotated ROIs by expert radiologists. Patients were excluded if they had the following: (I) missing image data; (II) poor DM image quality, (III) breast implants; (IV) a DM image that could not be segmented; or (V) incorrect image segmentation determined by a radiologist. Finally, a total of 186 patients were included in the study, grouped as follows: 58 luminal A, 35 luminal B, 52 TNBC, and 41 HER2. The images were translated into a total of 413 images, including cranio caudal (CC) and mediolateral oblique (MLO) views. Combining CC and MLO views has shown improved classification performance [18,19]. The included images were divided into a training set (70%) and a testing set (30%).

### 2.3. Tumor Segmentation

**Figure 2:**
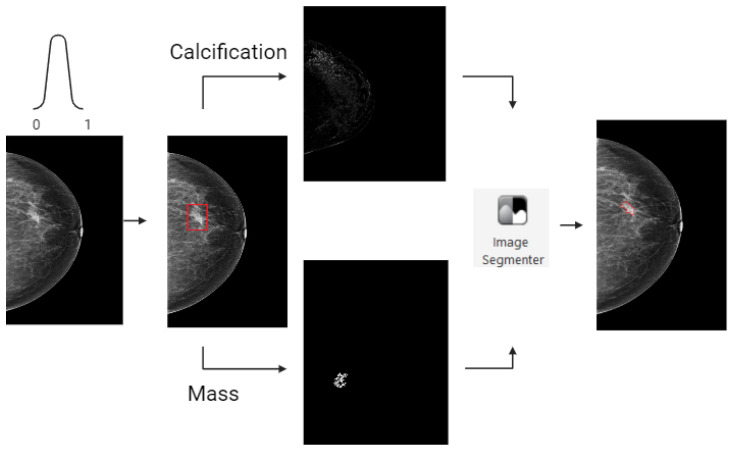
The tumor segmentation process. Starting with normalization of the original DM image, where breast lesions (red) classified as ‘calcification’ underwent image enhancement. Breast lesions classified as ‘mass’ underwent segmentation using a region-growing algorithm. The segmentations were finalized with the use of the image segmenter tool from MATLAB to obtain the final tumor segmentation.

**Figure 3:**
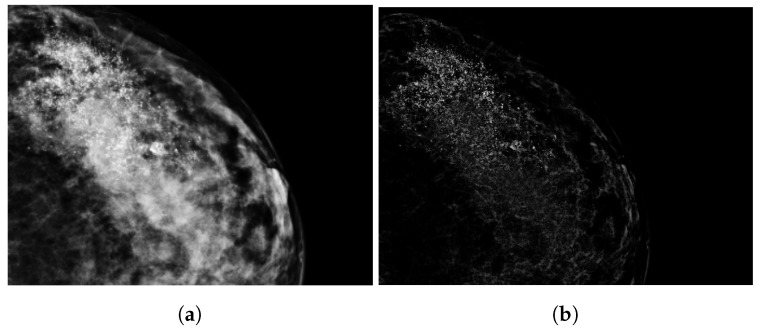
An example of image enhancement for a calcification region where (**a**) is the original DM image and (**b**) is the enhanced image, making the calcification more pronounced.

A visual presentation of the image segmentation process is shown in Figure 2. First, we normalized the DM images to a scale of [0, 1]. After this DM image normalization, breast lesions classified as ‘calcification’ underwent contrast enhancement, shown in Figure 3. This process highlighted variations in pixel intensities, increasing the intensities of calcification while minimizing those of surrounding breast tissue. Tumors classified as ‘mass’ underwent lesion segmentation through a semi-automatic approach using the region-growing algorithm [25]. The MaxDiff parameter was used to set the maximum allowable difference in average pixel intensity among the segmented pixels. For both segmentations, calcification and mass, the initial masks were refined in MATLAB (version R2023b) with the Image Segmenting tool, after which, an expert breast radiologist with over 30 years of experience reviewed 80 segmentations (19%) and confirmed, adjusted, or excluded these segmentations.

### 2.4. Radiomics Features

Radiomics features were extracted from the segmented images using Pyradiomics, an open-source Python package (https://pyradiomics.readthedocs.io/en/latest/, version 3.1.0, accessed on 31 July 2024). A total of 107 features were extracted: 14 shape-based features, 18 first-order statistics features, 24 gray-level co-occurrence matrix (GLCM) features, 16 gray-level run-length matrix (GLRLM) features, 16 gray-level size zone matrix (GLSZM) features, 14 gray-level distance zone matrix (GLDZM) features, and 5 neighborhood gray-tone difference matrix (NGTDM) features. Prior to their use in ML models, these features were normalized using z-score normalization.

Feature selection was performed using Pearson’s correlation and Least Absolute Shrinkage and Selection Operator (LASSO). Pearson’s correlation was used to pre-select features, and the correlation threshold was set to 0.8. If the correlation coefficient between two features exceeded this threshold, the feature with the highest mean absolute correlation coefficient was removed to eliminate redundancy. When employing LASSO, the hyperparameter lambda was tuned through ten-fold cross-validation. In cross-validation, the model was trained and evaluated 10 times, each with a different subset as the test set. Radiomic features with non-zero LASSO coefficients were selected for the model. Both naive Bayes (NB) and support vector machine (SVM) classifiers were employed for the classification tasks. During SVM classification, the optimal kernel (linear, sigmoid or radial) was determined, and the cost parameter was tuned through ten-fold cross-validation.

Our study focused on four binary classification tasks: (1) luminal A vs. non-luminal A, (2) luminal B vs. non-luminal B, (3) TNBC vs. non-TNBC, and (4) HER2 vs. non-HER2. The dataset was randomly divided into a training set (70%) and a testing set (30%). To overcome the problem of class imbalance in the training set, we applied the Synthetic Minority Oversampling Technique (SMOTE), as was performed in previous studies addressing the same problem for class imbalance [18,20]. SMOTE combines undersampling of the majority class with oversampling of the minority class. The percentage of oversampling was set to 100% (balancing the training data to a 50–50% split).

### 2.5. Statistical Analysis

Features selected by Pearson’s correlation were evaluated for statistical significance (*p* < 0.05) using the Kruskal–Wallis test. Classification performance was assessed by area under receiver operating characteristic curve (AUC) and accuracy. DeLong’s test was used to compare the AUCs between the SVM and NB classifier.

All feature selection, model building, and statistical analysis were performed in Rstudio (version 2023.09.0).

## 3. Results

The patients’ ages ranged from 47 to 79 years, with an average age at screening of 59.6 ± 6.9. The age variable was not tested for normality. A one-way ANOVA test showed no statistically significant differences in age between the groups (*p* > 0.05).

### 3.1. Radiomic Features

**Figure 4:**
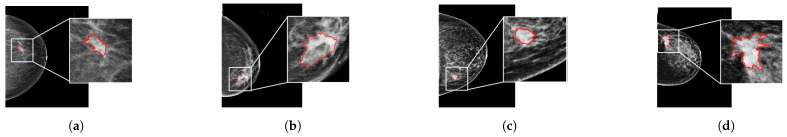
Examples of breast tumor segmentations for (**a**) luminal A, (**b**) luminal B, (**c**) TNBC, and (**d**) HER2.

**Figure 5:**
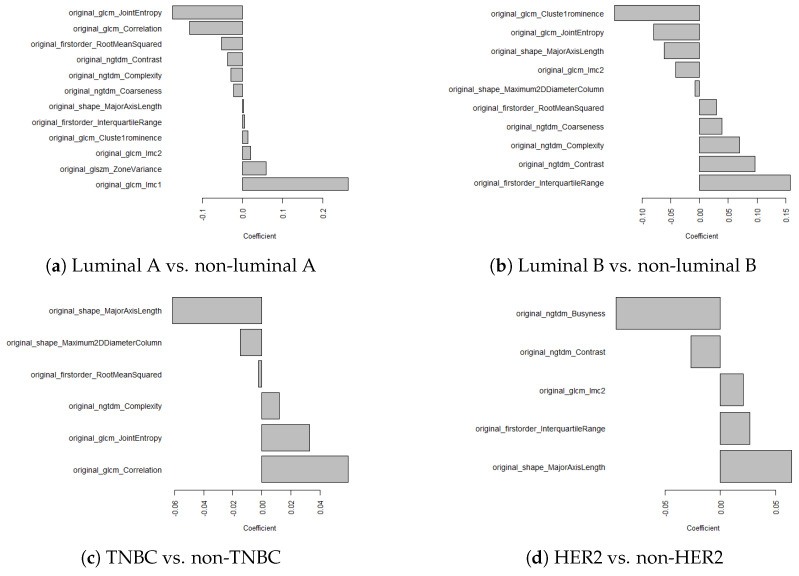
The selected radiomic features for (**a**) luminal A vs. non-luminal A, (**b**) luminal B vs. non-luminal B, (**c**) TNBC vs. non-TNBC, and (**d**) HER2 vs. non-HER2 classification tasks.

The radiomic features were extracted based on the tumor segmentations, with examples shown in Figure 4. Two radiomic features, namely original_shape_Flatness and original_shape_LeastAxisLength, were excluded due to yielding zeros during the feature extraction process. Pearson correlation pre-identified 13, 14, 14, and 16 features for luminal A, luminal B, TNBC, and HER2, respectively. All 16 features pre-selected by Pearson correlation showed statistically significant differences between the subtypes. After using LASSO for further feature selection, this resulted in a final set of 12, 10, 6, and 5 features for luminal A, luminal B, TNBC, and HER2, respectively. The selected features and corresponding LASSO coefficients are depicted in Figure 5.

### 3.2. Classification Performance

**Figure 6:**
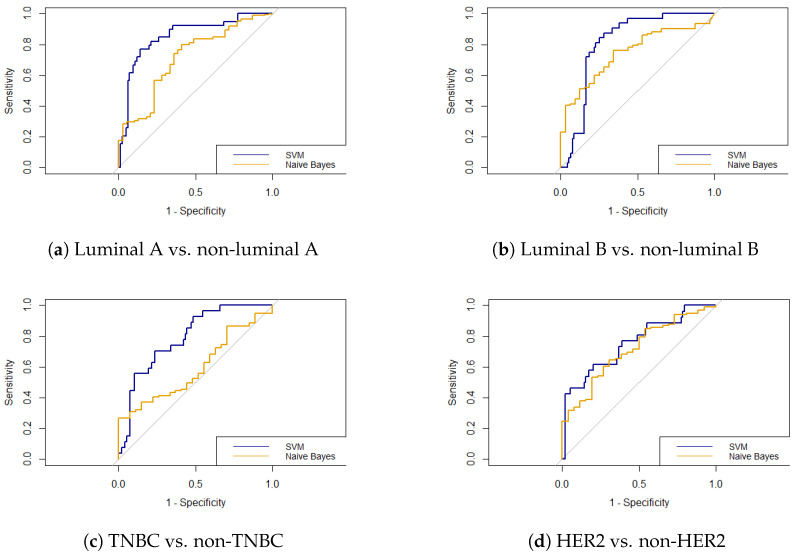
The ROC curves of the SVM (blue) and NB (yellow) classifiers for (**a**) luminal A vs. non-luminal A, (**b**) luminal B vs. non-luminal B, (**c**) TNBC vs. non-TNBC, and (**d**) HER2 vs. non-HER2.

Table 3 shows the classification performance of the SVM and NB classifiers for the testing set. As can be seen, AUCs in SVM classification ranged from 0.755 to 0.855, while AUCs in NB classification ranged from 0.593 to 0.714. The corresponding ROC curves are depicted in Figure 6. DeLong’s test revealed that the AUCs from SVM classification were higher compared to the NB classifier, and statistically significant differences were observed for luminal A (*p* = 0.027) and TNBC (*p* = 0.007). There were no significant differences found for the luminal B (*p* = 0.273) and HER2 (*p* = 0.596) classification. The accuracy ranged from 0.581 to 0.815 for SVM classification and ranged from 0.484 to 0.750 for NB classification. In SVM classification, higher accuracies were observed for luminal A and TNBC. For luminal B and HER2 classification, the NB classifier showed greater accuracies.

## 4. Discussion

The aim of this study was to explore the potential for non-invasively predicting breast cancer subtypes using screening DM images. Four binary classification tasks (luminal A vs. non-luminal A, luminal B vs. non-luminal B, TNBC vs. non-TNBC, and HER2 vs. non-HER2) were designed, and two ML classifiers were used, the SVM and NB classifiers.

Previous studies showed the associations between DM image characteristics and breast cancer subtypes. S. Taneja [26] described that HER2 and TNBC tumors tend to show more indistinct margins and fewer spiculated lesions on DM images, unlike luminal A and luminal B subtypes, which are more likely to show spiculated lesions. M. Boisserie-Lacroix [27] also explored the relationship between DM images and breast cancer subtype characteristics. Similar to S. Taneja, their study revealed that luminal A and luminal B tend to show more spiculated masses with irregular shapes. Further, M. Boisserie-Lacroix described HER2 lesions to be irregular in shape with indistinct margins, while TNBC lesions were more often oval in shape with distinct edges. A comprehensive investigation of the associations between breast cancer subtypes and their corresponding radiomic features was performed. Pearson correlation pre-identified features for the classification of each molecular subtype, and it is worth mentioning that all features showed statistical significance between the subtypes.

The results of the feature selection showed that the zone variance was identified as an important feature for luminal A classification. The zone variance feature describes the roughness of the tumoral edges as well as the strength feature that describes tumor margins. Our findings revealed that values of zone variance were highest in HER2, which indicated that HER2 may present rougher edges and unclear margins. This finding was in line with previous research that reported higher zone variance values for HER2 tumors [23]. On the other hand, the lowest values were found in TNBC, suggesting clearer and more well-defined margins, aligning with Biosserie-Lacroix’s study [27]. Strength was selected for both luminal A and luminal B classification, where luminal B reached the highest values. This result suggests that luminal B tumors tend to be associated with unclear margins, confirming Boisserie-Lacroix and S. Niu’s studies [23,27].

Regarding the heterogeneity of the tumor, the features coarseness, contrast, and correlation were selected. Coarseness describes the differences in the gray level between the central pixel and surrounding area, with high values indicating a more homogeneous lesion [10]. The values of this feature were highest for luminal B and lowest for TNBC; thus, luminal B tends to be more homogeneous and TNBC more heterogeneous. In our work, ngtdm_Contrast retrieved the highest values for luminal B, suggesting luminal B tumors to be more homogeneous compared to the other subtypes, which is in accordance with L. Nicosa’s study [28]. The correlation feature describes the relationship between gray levels of neighboring pixels. This feature was selected for luminal A and TNBC classification, with higher correlation values suggesting a homogeneous lesion. W. Ma’s study [18] showed the highest correlation values for luminal lesions; however, during our study, the highest values were shown in TNBC lesions. Though the coarseness and contrast features are in line with W. Ma’s study, the correlation features suggest that TNBC tumors may be smoother in texture compared to the other subtypes.

A feature that describes the shape of the tumor which was selected for each binary classification was MajorAxisLength. Higher MajorAxisLength values suggesting tumors to be rounder in shape. Our results showed that HER2 lesions exhibited the highest values and TNBC the lowest for MajorAxisLength. This suggests that HER2 tumors tend to be rounder in shape, in contrast with TNBC tumors that may be less round. While several articles describe TNBC tumors to be rounder in shape [10,18], others report TNBC tumors to be larger and irregular in shape [29].

These varying descriptions of tumor shape and tumor heterogeneity characteristics associated with breast cancer subtypes in the existing literature, as well as the differing results in our study, indicate that further research is necessary to clarify the characteristic associated with breast cancer subtypes.

Most previous studies on the prediction of breast cancer subtypes or breast cancer risk focused on using one ML classifier. During our study, two commonly used ML classifiers were used, and their performance was compared: the SVM, which is mainly used for classifying multidimensional data, and NB classification, which is a relatively simple method that calculates probabilities to make predictions [30,31]. The SVM classifier resulted in higher AUC during testing compared to the NB classifier. There was only statistical significance between the SVM and NB for luminal A (*p* = 0.0268) and TNBC (*p* = 0.0073). The study of N. Mao [32] found similar results, with the SVM outperforming NB in distinguishing benign and malignant tumors.

Our study has presents some limitations. One of these limitations is the image segmentation process. Although we were given the exact location of the lesion in the form of a rectangular ROI, the segmentations were carried out by one (inexperienced) person. Since radiomic features are highly influenced by the segmentation, this introduces a potential bias with regards to the extracted features. On the other hand, our study applied the RegGrow algorithm, which is a semi-automatic segmentation approach. This approach offers a notable advantage since it is less time-consuming and less subjective compared to manual segmentation.

Additionally, while the current accuracy of the radiomics-based approach is under 80%, it is important to highlight that this technique is currently a complementary tool rather than a replacement for CNB in this stage. At present, radiomics could be used in conjunction with biopsy results to improve the decision-making by narrowing down the subtype classification before more invasive procedures are performed, ultimately reducing the number of unnecessary biopsies.

Furthermore, only a portion of the segmented lesions was checked by an expert radiologist. The lack of inter-observer reproducibility assessment may introduce an observer bias. Differences in tumor segmentation can lead to variations in feature extraction for similar areas of interest, as was mentioned in previous studies [33,34,35]. To address these limitations, it should be ensured that all the segmented regions are revised and adjusted by an expert radiologist in related future work. Subsequently, feature extraction should be repeated to verify the results.

Moreover, this was a retrospective study, and the DM images were from a single dataset. It is important to verify the performance obtained during this study using an external dataset to confirm generalizability of the models. Also, the current results are a first step, and with relatively low accuracies, our research indicates that there is significant room for further improvements. For example, even larger datasets and ensemble machine learning methods can be incorporated.

Currently, there is a lack of consensus on the optimal ML classifier for predicting breast cancer subtypes, and limited studies compare the performance of various classifiers. Our study focused on two ML classifiers; however, future research should explore and compare more classifiers such as kNN, Random Forest, and logistic regression. By evaluating these classifiers’ performance, more insight into the most effective method for the prediction of breast cancer subtypes can be gained. This could enhance the generalizability and utility of radiomic features in predicting breast cancer subtypes, potentially laying the groundwork for a robust radiomic workflow in this field.

DM is currently the most commonly used technique in breast cancer screening. However, DBT has also gained attention in routine screening and has been shown to improve screening performance when compared to DM [36]. The study of S. Nui explored different imaging modalities for the diagnosis of breast cancer, including DM, DBT, and MRI [37]. Standalone DBT images achieved higher AUCs in validation compared to the AUC from standalone DM images, yet the study showed that the combination of DM and DBT features in a model significantly improved the performance. The OMI-DB database that was used includes over 2000 DBT cases, each linked to their corresponding DM images. Future research could look into the potential of combining DM and DBT into a model and comparing these results to the current DM-only models to check if combining these modalities increases the model efficacy.

The rise of larger databases and greater computing power has increased interest in other AI algorithms based on deep learning and convolutional neural networks (CNNs). In fact, there are some recently published works addressing the use of these type of AI in predicting the breast cancer molecular subtype in mammography images [20,21,22,38]. Three of them focus on a binary classification for just one molecular subtype [20,21,22], while another applies data balancing techniques to classify all molecular types [38]. As for the latter, which is the first study using CNNs to classify the tumor considering all the subtypes, although the focus was on the data balancing method, the AUCs obtained for each subtype are all lower than those we obtained in our study. Deep learning/CNN algorithms do not require precise segmentation, only a region of interest containing the lesion, thus eliminating the need for the important segmentation step. However, this segmentation of the tumors, extraction of their features, and analysis using conventional statistical and machine learning algorithms give us a much deeper understanding of what we are studying, in some cases making it easier to understand how the models behave in a certain way.

## 5. Conclusions

In conclusion, our study suggests that quantitative image features extracted from DM images show the potential to contribute to the classification of breast cancer subtypes. Although the SVM classifier showed better performance compared to the NB classifier, the overall accuracies are still sub-optimal. Our findings show that while radiomic features are associated with breast cancer characteristics, the current model’s performance is not yet sufficient for standalone clinical use. Further research is warranted to validate these models using external datasets and assess their generalizability. Additionally, exploring the combination of DM and DBT radiomic features could potentially improve its performance.

## Figures and Tables

**Table 1:** Overview of research on breast cancer subtype predictions with the use of radiomics.

	Technique	Purpose	Findings
W. Ma [18]	DM	Luminal vs. non-luminal TNBC vs. non-TNBC HER2 vs. non-HER2	TNBC was differentiated from non-TNBC with an AUC/accuracy of 0.865/0.796. HER2 could be distinguished with an AUC/accuracy of 0.784/0.748 and the luminal type 0.752/0.788
J. Son [13]	Synthetic DM	Luminal vs. non-luminal TNBC vs. non-TNBC HER2 vs. non-HER2	The AUC, accuracy, sensitivity, and specificity for the TNBC model were 0.838, 0.803, 0.833, and 0.797. For HER2, this resulted in values of 0.556, 0.704, 0.111, and 0.790, respectively. When distinguishing the luminal subtype, AUC, accuracy, sensitivity, and specificity values of 0.645, 0.507, 0.440, and 0.667 were obtained.
J. Zhou [19]	DM	HER2 vs. non-HER2	The SVM classifier resulted in AUC, accuracy, sensitivity, and specificity values of 0.740, 0.730, 0.688, and 0.609. The logistic regression model resulted in AUC/ACC/SENS/SPEC of 0.787/0.770/0.688/0.739.
Y. Deng [20]	DM	HER2 vs. non-HER2	The AUC and accuracy of distinguishing HER2 vs. non-HER2 was 0.776 and 0.712 during testing. In the external validation set, the AUC and accuracy was 0.702 and 0.700.
L. Wang [21]	DM	TNBC vs. non-TNBC	Accuracy, sensitivity, and specificity values of 0.84, 0.81, and 0.78, respectively, were obtained.
Y. Zhang [14]	CESM	TNBC vs. non-TNBC	Resulted in AUC, sensitivity, and specificity values of 0.90, 0.97, and 0.69.
A. Petrillo [15]	CESM	HER2 vs. non-HER2	Tested accuracies, sensitivities, and specificities for the logistic regression, CART, and Random Forest models. A combination of features from CC and MLO showed the highest accuracies of > 90% using a classification tree algorithm. For HER2 classification, the best accuracies were obtained with an RF algorithm.
D. La Forgia [16]	CESM	Histological outcome	Resulted in AUC values of ER+/ER−: 0.838, PR+/PR−: 0.755, Ki67+/Ki67−: 0.848, high-grade/low-grade: 0.799, TNBC/NTNBC: 0.768, and HER2/HER2−: 0.909.
S. Zhu [17]	CESM	Luminal vs. non-luminal TNBC vs. non-TNBC HER2 vs. non-HER2	Showed AUC values during combined low energy and recombined images during testing for luminal, HER2, and TNBC values of 0.93, 0.89, and 0.87, respectively. For the external dataset, this resulted in AUC values of 0.82, 0.83, and 0.68 for luminal, HER2, and TNBC, respectively.
S. Niu [23]	DM, DBT, and MRI	Intra- and peritumoral regions	AUC values for distinguishing luminal A, luminal B, HER2, and TNBC of 0.762, 0.757, 0.756, and 0.771 were obtained for DM images.
S. GE [22]	DM	TNBC vs. non-TNBC	Distinguishing TNBC vs. non-TNBC resulted in AUC, accuracy, sensitivity, and specificity values of 0.809, 0.806, 0.720, and 0.801.

**Table 2:** Immunohistochemical (IHC) expression and the subtype classification scheme applied during this study.

	ER	PR	HER2
Luminal A	+	+	−
Luminal B	+	+/−	−
TNBC	−	−	−
HER2	−	−	+

**Table 3:** Classification performance for SVM and NB classification tasks for the testing set.

	SVM		NB	
	Accuracy	AUC	Accuracy	AUC
		(95%-CI)		(95%-CI)
Luminal A	0.815	0.855	0.726	0.714
		(0.779–0.930)		(0.616–0.812)
Luminal B	0.734	0.812	0.750	0.746
		(0.736–0.889)		(0.655–0.837)
TNBC	0.581	0.789	0.484	0.593
		(0.701–0.878)		(0.482–0.704)
HER2	0.637	0.755	0.718	0.714
		(0.644–0.867)		(0.608–0.819)

## Data Availability

The dataset presented in this article is not readily available. Requests to access the datasets should be directed to the OPTIMAM providers.

## Abbreviations

The following abbreviations are used in this manuscript:

AUC	Area Under the Curve
CESM	Contrast-enhanced spectral mammography
CC	Cranio caudal
CNB	Core needle biopsy
DBT	Digital Breast Tomosynthesis
DM	Digital mammography
ER	Estrogen receptor
HER	Human epidermal growth factor receptor
IHC	Immunohistochemical
LASSO	Least Absolute Shrinkage and Selection Operator
ML	Machine learning
MLO	Medio-lateral oblique
MRI	Magnetic Resonance Imaging
NB	Naive Bayes
OMI-DB	OPTIMAM Mammography Image Database
PR	Progesteron receptor
ROC	Receiver Operating Curve
ROI	Region of interest

SMOTE	Synthetic Minority Oversampling Technique
SVM	Support vector machine
TNBC	Triple-negative breast cancer

## References

[1] Siegel R.L., Giaquinto A.N., Jemal A. (2024). Cancer statistics, 2024. CA A Cancer J. Clin..

[2] Sung H., Ferlay J., Siegel R.L., Laversanne M., Soerjomataram I., Jemal A., Bray F. (2021). Global Cancer Statistics 2020: GLOBOCAN Estimates of Incidence and Mortality Worldwide for 36 Cancers in 185 Countries. CA A Cancer J. Clin..

[3] WHO (2022). A Short Guide to Cancer Screening: Increase Effectiveness, Maximize Benefits and Minimize Harm.

[4] Harbeck N., Penault-Llorca F., Cortés J., Gnant M., Houssami N., Poortmans P., Ruddy K., Tsang J., Cardoso F. (2019). Breast cancer. Nat. Rev. Dis. Primers.

[5] Orrantia-Borunda E., Anchondo-Nuñez P., Acuña-Aguilar L.E., Gómez-Valles F.O., Ramírez-Valdespino C.A. (2022). Subtypes of Breast Cancer. Breast Cancer.

[6] Phipps A.I., Li C.I., Li C. (2010). Breast Cancer Biology and Clinical Characteristics. Breast Cancer Epidemiology.

[7] Bilous M. (2010). Breast core needle biopsy: Issues and controversies. Mod. Pathol..

[8] Panet F., Papakonstantinou A., Borrell M., Vivancos J., Vivancos A., Oliveira M. (2024). Use of ctDNA in early breast cancer: Analytical validity and clinical potential. NPJ Breast Cancer.

[9] Sant M., Bernat-Peguera A., Felip E., Margelí M. (2022). Role of ctDNA in Breast Cancer. Cancers.

[10] Mayerhoefer M.E., Materka A., Langs G., Häggström I., Szczypiński P., Gibbs P., Cook G. (2020). Introduction to Radiomics. J. Nucl. Med..

[11] Van Timmeren J., Cester D., Tanadini-Lang S., Alkadhi H., Baeßler B. (2020). Radiomics in medical imaging—“How-to” guide and critical reflection. Insights Imaging.

[12] Panico A., Gatta G., Salvia A., Grezia G.D., Fico N., Cuccurullo V. (2023). Radiomics in Breast Imaging: Future Development. J. Pers. Med..

[13] Son J., Lee S.E., Kim E.K., Kim S. (2020). Prediction of breast cancer molecular subtypes using radiomics signatures of synthetic mammography from Digital Breast Tomosynthesis. Sci. Rep..

[14] Zhang Y., Liu F., Zhang H., Ma H., Sun J., Zhang R., Song L., Shi H. (2021). Diagnostic value of radiomics analysis in contrast-enhanced spectral mammography for identifying triple-negative breast cancer. Front. Oncol..

[15] Petrillo A., Fusco R., Bernardo E., Petrosino T., Barretta M., Porto A., Granata V., Bonito M., Fanizzi A., Massafra R. (2022). Prediction of Breast Cancer Histological Outcome by Radiomics and Artificial Intelligence Analysis in Contrast-Enhanced Mammography. Cancers.

[16] Forgia D., Fanizzi A., Campobasso F., Bellotti R., Didonna V., Lorusso V., Moschetta M., Massafra R., Tamborra P., Tangaro S. (2020). Radiomic Analysis in Contrast-Enhanced Spectral Mammography for Predicting Breast Cancer Histological Outcome. Diagnostics.

[17] Zhu S., Wang S., Guo S., Wu R., Zhang J., Kong M., Pan L., Gu Y., Yu S. (2023). Contrast-enhanced mammography radiomics analysis for preoperative prediction of breast cancer molecular subtypes. Acad. Radiol..

[18] Ma W., Zhao Y., Ji Y., Guo X., Jian X., Liu P., Wu S. (2019). Breast Cancer Molecular Subtype Prediction by Mammographic Radiomic Features. Acad. Radiol..

[19] Zhou J., Tan H., Bai Y., Li J., Lu Q., Chen R., Zhang M., Feng Q., Wang M. (2019). Evaluating the HER-2 status of breast cancer using mammography radiomics features. Eur. J. Radiol..

[20] Deng Y., Lu Y., Li X., Zhu Y., Zhao Y., Ruan Z., Mei N., Yin B., Liu L. (2024). Prediction of human epidermal growth factor receptor 2 (HER2) status in breast cancer by mammographic radiomics features and clinical characteristics: A multicenter study. Eur. Radiol..

[21] Wang L., Yang W., Xie X., Liu W., Wang H., Shen J., Ding Y., Zhang B., Song B. (2020). Application of digital mammography-based radiomics in the differentiation of benign and malignant round-like breast tumors and the prediction of molecular subtypes. Gland. Surg..

[22] Ge S., Yixing Y., Jia D., Ling Y. (2022). Application of mammography-based radiomics signature for preoperative prediction of triple-negative breast cancer. BMC Med. Imaging.

[23] Niu S., Jiang W., Zhao N., Jiang T., Dong Y., Luo Y., Yu T., Jiang X. (2022). Intra- and peritumoral radiomics on assessment of breast cancer molecular subtypes based on mammography and MRI. J. Cancer Res. Clin. Oncol..

[24] Halling-Brown M.D., Warren L.M., Ward D., Lewis E., Mackenzie A., Wallis M.G., Wilkinson L.S., Given-Wilson R.M., McAvinchey R., Young K.C. (2021). Optimam Mammography Image Database: A large-scale resource of mammography images and Clinical Data. Radiol. Artif. Intell..

[25] Wisselink H.J. (2024). RegGrow. https://github.com/thrynae/RegGrow/releases/tag/v1.3.0.

[26] Taneja S., Evans A.J., Rakha E.A., Green G., Ellis I.O. (2008). The mammographic correlations of a new immunohistochemical classification of invasive breast cancer. Clin. Radiol..

[27] Boisserie-Lacroix M., Hurtevent-Labrot G., Ferron S., Lippa N., Bonnefoi H., Mac Grogan G. (2013). Correlation between imaging and molecular classification of breast cancers. Diagn. Interv. Imaging.

[28] Nicosia L., Bozzini A., Ballerini D., Palma S., Pesapane F., Raimondi S., Gaeta A., Bellerba F., Origgi D., De Marco P. (2022). Radiomic Features Applied to Contrast Enhancement Spectral Mammography: Possibility to Predict Breast Cancer Molecular Subtypes in a Non-Invasive Manner. Int. J. Mol. Sci..

[29] Li H., Zhu Y., Burnside E., Huang E., Drukker K., Hoadley K., Fan C., Conzen S., Zuley M., Net J. (2016). Quantitative MRI radiomics in the prediction of molecular classifications of breast cancer subtypes in the TCGA/TCIA data set. npj Breast Cancer.

[30] Jalloul R., Chethan H., Alkhatib R. (2023). A Review of Machine Learning Techniques for the Classification and Detection of Breast Cancer from Medical Images. Diagnostics.

[31] Berrar D. (2018). Bayes’ Theorem and Naive Bayes Classifier. Ref. Modul. Life Sci..

[32] Mao N., Yin P., Wang Q., Liu M., Dong J., Zhang X., Xie H., Hong N. (2019). Added Value of Radiomics on Mammography for Breast Cancer Diagnosis: A Feasibility Study. J. Am. Coll. Radiol..

[33] Scapicchio C., Gabelloni M., Barucci A., Cioni D., Saba L., Neri E. (2021). A deep look into radiomics. La Radiol. Medica..

[34] Haarburger C., Müller-Franzes G., Weninger L., Kuhl C., Truhn D., Merhof D. (2020). Radiomics feature reproducibility under inter-rater variability in segmentations of CT images. Sci. Rep..

[35] Conti A., Duggento A., Indovina I., Guerrisi M., Toschi N. (2021). Radiomics in breast cancer classification and prediction. Semin. Cancer Biol..

[36] Lowry K., Coley R., Miglioretti D., Kerlikowske K., Henderson L., Onega T., Sprague B., Lee J., Herschorn S., Tosteson A. (2020). Screening Performance of Digital Breast Tomosynthesis vs. Digital Mammography in Community Practice by Patient Age, Screening Round, and Breast Density. JAMA Netw. Open.

[37] Niu S., Wang X., Zhao N., Liu G., Kan Y., Dong Y., Cui E.N., Luo Y., Yu T., Jiang X. (2021). Radiomic Evaluations of the Diagnostic Performance of DM, DBT, DCE MRI, DWI, and Their Combination for the Diagnosisof Breast Cancer. Front. Oncol..

[38] Mota A., Mendes J., Matela N. (2024). Breast Cancer Molecular Subtype Prediction: A Mammography-Based AI Approach. Biomedicines.

